# High Prevalence and Early Occurrence of Skeletal Complications in EGFR Mutated NSCLC Patients With Bone Metastases

**DOI:** 10.3389/fonc.2020.588862

**Published:** 2020-11-12

**Authors:** Marta Laganà, Cristina Gurizzan, Elisa Roca, Diego Cortinovis, Diego Signorelli, Filippo Pagani, Anna Bettini, Lucia Bonomi, Silvia Rinaldi, Rossana Berardi, Marco Filetti, Raffaele Giusti, Sara Pilotto, Michele Milella, Salvatore Intagliata, Alice Baggi, Alessio Cortellini, Hector Soto Parra, Matteo Brighenti, Fausto Petrelli, Chiara Bennati, Paolo Bidoli, Marina Chiara Garassino, Alfredo Berruti

**Affiliations:** ^1^ Medical Oncology, Department of Medical and Surgical Specialties, Radiological Sciences and Public Health University of Brescia, ASST-Spedali Civili, Brescia, Italy; ^2^ Medical Oncology, Ospedale S. Gerardo di Monza, Monza, Italy; ^3^ Medical Oncology, Fondazione IRCCS Istituto Nazionale Tumori, Milano, Italy; ^4^ Medical Oncology, ASST Papa Giovanni XXIII di Bergamo, Bergamo, Italy; ^5^ Medical Oncology, Ospedali Riuniti di Ancona, Ancona, Italy; ^6^ Medical Oncology, Azienda Ospedaliero Universitaria S. Andrea di Roma, Roma, Italy; ^7^ Medical Oncology, Università degli studi di Verona, Azienda Ospedaliera Universitaria Integrata, Verona, Italy; ^8^ Medical Oncology, Ospedale San Salvatore di L’Aquila, L’Aquila, Italy; ^9^ Medical Oncology, Policlinico Vittorio Emanuele di Catania, Catania, Italy; ^10^ Medical Oncology, Ospedale di Cremona, Cremona, Italy; ^11^ Ospedale Treviglio, ASST Bergamo Ovest, Treviglio, Italy; ^12^ Ospedale Santa Maria delle Croci di Ravenna, Ravenna, Italy

**Keywords:** bone metastasis, non-small cell lung cancer, skeletal related events, tyrosine kinase inhibitors, epidermal growth factor receptor

## Abstract

**Objectives:**

The prevalence of Skeletal Related Adverse Events (SREs) in EGFR mutated non-small cell lung cancer (NSCLC) patients with bone metastases, treated with modern tyrosine kinase inhibitors (TKIs), has been scarcely investigated.

**Materials and Methods:**

We retrospectively evaluated the data of EGFR mutated NSCLC patients with bone metastases treated with TKIs in 12 Italian centers from 2014 to 2019, with the primary aim to explore type and frequency of SREs.

**Results:**

Seventy-seven out of 274 patients enrolled (28%) developed at least one major SRE: 55/274 (20%) bone fractures, 30/274 (11%) spinal cord compression, 5/274 (2%) hypercalcemia. Median time to the onset of SRE was 3.63 months. Nine patients (3%) underwent bone surgery and 150 (55%) radiation therapy on bone. SREs were more frequently observed within the 12 months from TKI start than afterwards (71 *vs* 29%, p 0.000). Patient Performance Status and liver metastases where independently associated with the risk of developing SREs. Median TKI exposure and overall survival were 11 and 28 months, respectively. Bone resorption inhibitors were associated with a lower risk of death (HR 0.722, 95% CI: 0.504–1.033, p = 0.075) although not statistically significant at multivariate analysis.

**Conclusion:**

Bone metastatic NSCLC patients with EGFR mutated disease, treated with EGFR TKIs, have a relatively long survival expectancy and are at high risk to develop SREs. The early SRE occurrence after the TKI start provides the rationale to administer bone resorption inhibitors.

## Introduction

Lung cancer is the leading cause of cancer death worldwide (1).

Despite the introduction of modern efficacious therapies, the prognosis of patients with metastatic disease still remains poor although highly variable, being dependent on genomic abnormalities and programmed death-ligand 1 (PD-L1) expression (2).

Genetic analysis allows the identification of somatic sensitizing mutations in epidermal growth factor receptor (EGFR), typically exon 19 deletion (Ex19del) and L858R. These mutations are found in among 15% of lung adenocarcinoma in European patients (2).

First- or second-generation EGFR tyrosine kinase inhibitors (EGFR-TKIs) (e.g., erlotinib, gefitinib, and afatinib), administered to patients whose tumors harbor these genotype alterations, led to marked tumor response and improved progression-free survival and quality of life over chemotherapy (3–5). Thanks to these efficacious drugs the overall survival of patients with activating EGFR mutations has increased from a median of 7.9 months in 2002 to 27.3 months in 2015 (6).

The prognosis of these patients is destined to further improve with the recent introduction in clinics of the third generation EGFR-TKI osimertinib (7).

NSCLC often metastasizes to bone and the frequency of bone metastasis (BM) is 30–40% during the clinical course of the disease (8).

The diagnosis of BM negatively impacts on patient’s quality of life (QoL) and is associated with poor survival (9). About 80% of bone metastatic lung cancer patients experience significant pain and more than 60% develop skeletal-related events (SREs), usually defined as bone surgery, bone radiation therapy, pathological fractures, spinal cord compression, and hypercalcemia (9).

SREs is therefore a composite endpoint, which encompasses both major complications, such as fractures, spinal cord compression, hypercalcemia, and local bone treatments, such as surgery and radiation therapy (10).

Published randomized clinical trials did not report whether the greater benefit obtained by modern TKIs over chemotherapy could translate or not into a fewer proportion of SREs (11).

The therapeutic strategies to manage BM and reduce the incidence of SREs include the administration of bisphosphonates (i.e. zoledronic acid) and Receptor Activator of Nuclear factor Kappa B Ligand (RANKL) inhibitors (denosumab). The results of a randomized prospective placebo controlled clinical trial have demonstrated that zoledronic acid is efficacious in preventing and delaying the SRE onset in lung cancer patients with bone metastases (10). In the same patient population, a subsequent prospective phase III randomized clinical trial demonstrated the superiority of denosumab over zoledronic acid in terms of prevention and delay of SREs (12). These randomized clinical trials were conducted before the introduction of TKIs in the management of lung cancer.

In this paper, we present the results of a retrospective multicenter study aiming to define the prevalence of SREs in EGFR mutated NSCLC patients, treated with first/second generation TKIs.

We also investigated the natural history of EGFR mutated NSCLC with BM, the prognostic factors, and the impact of bone resorption inhibitors on patient’s outcome.

## Patients and Methods

### Study Design

We conducted a retrospective, observational, multicenter study. Medical records of patients with bone metastases from EGFR-mutated NSCLC, treated with TKIs in 12 referral Italian centers from 2014 to 2019, were analyzed.

The primary aim of the study was to estimate the frequency of SREs. Secondary endpoints were to define in this study population overall survival, time to TKIs exposure and time to SRE. We also explored, both in univariate and multivariate analyses, prognostic factors, and factors predictive of SREs.

To be included in our study adult patients (≥18 years) with EGFR-mutated lung adenocarcinoma should have had bone metastatic involvement either at diagnosis or during the disease course (synchronous *versus* metachronous bone metastases) and identified through imaging assessment (e.g. standard x-rays, computed tomography scans, magnetic resonance imaging, or 18fluoro-deoxy-glucose positron-emission tomography of the skeleton). Patients with bone invasion by contiguity were excluded.

Clinical data were collected in an anonymized database, including demographic data such as age at cancer diagnosis, gender, Performance Status (PS) according to ECOG, major comorbidities, current or past smoking history, site and number of visceral metastases. We also recorded pathological data, including EGFR mutations, and blood chemistry data: i.e. alkaline phosphatase (ALP), lactate dehydrogenase (LDH), hemoglobin (HB).

For each patient, the type of TKI used was collected, specifying date of start and end of treatment. Dates of disease progression under TKI therapy (defined according Response Evaluation Criteria in Solid Tumors [RECIST] version 1.1), last follow-up, or death were also recorded.

Regarding bone involvement, the following data were collected: time of onset of BMs, if BMs were synchronous or metachronous with respect to first disease diagnosis; if they were osteolytic, osteoblastic, or mixed; and the number of bone sites involved. Furthermore, data about the occurrence of SREs, i.e. pathological fractures, hypercalcemia, spinal cord compression, bone radiotherapy, and bone surgery, were collected as well as whether a specific bone anti-resorptive treatment (bisphosphonates or denosumab) was introduced or not.

The study was firstly approved by the Institutional Review Board of the Coordinating Center in Brescia (SURMOS Study no. NP1848) and subsequently by the ethic committees of each participating institution.

SURMOS study was conducted in accordance with the Declaration of Helsinki for clinical studies.

### Statistical Analysis

The primary endpoint was to define the prevalence of major SREs (fractures, spinal cord compression, and hypercalcemia) in EGFR mutated NSCLC patients with bone metastases, treated with TKIs.

Among secondary endpoints, we evaluated: 1) the prevalence of SREs according to standard definition (including also bone surgery and bone radiation therapy), 2) overall survival, that was defined as the time interval between the date of diagnosis of bone metastases and the date of death or the last known alive date, 3) time between primary diagnosis and occurrence of bone metastases, 4) time to the occurrence of SRE from the diagnosis of bone metastases, i.e. the interval between date of diagnosis of bone metastases to the first occurrence of either bone fracture, spinal cord compression, or hypercalcemia, and 5) time of TKI exposure (from the start to the end of TKI treatment, even beyond disease progression).

Descriptive statistics were used for patients’ demographics, tumor characteristics, and frequency of SREs. Categorized variables were expressed as percentages. Cut-off points were identified for continuous variables based on the median value or upper limit of normal ranges (for biochemical parameters).

Survival curves were calculated by the Kaplan-Meier method and differences compared by the log-rank test. The Cox’s proportional hazards regression model was employed to assess the Hazard ratios (HRs) and 95% confidence intervals (95% CIs) both in univariate and multivariate analyses, with the lowest risk group as the reference group. Only parameters significantly associated with OS or time to SRE in the univariate analysis (at p < 0.10) were included in the multivariate analysis model.

All p values are two-sided and p values less than 0.05 were considered statistically significant. Due to the explorative nature of this study, a formal calculation of the sample size was not performed. It was considered, however, that a minimum of 200 patients would have been required to have an adequate power for statistical analyses. Statistical Package for Social Science, SPSS, software (version 23.00, Chicago, IL, USA) was used for statistical analysis.

## Results

### Patient Characteristics and Treatment Administered

A total of 274 patients with bone metastatic, EGFR-mutated, NSCLC were collected to this study. Patient’s characteristics are summarized in **Table 1**.

**Table 1:** Patient’s characteristics.

Patients number (274)	Characteristics	Number (%)
**Gender**	Female Male	171 (62) 103 (38)
**Age at diagnosis**	≥50 yrs <50 yrs	247 (90) 27 (10)
**Smoking status**	Yes No/Unknown	182 (66) 92 (34)
**Performance status**	0–1 <1 Missing	222 (91) 21 (9) 31
**EGFR mutation**	18/20 19 21 Missing	22 (9) 144 (57) 85 (34) 23
**EGFR mutation T790M**	Yes No Missing	28 (55) 23 (45) 51
**Number of metastatic sites**	Only bone mets 2 mets ≥3	12 (4) 36 (13) 226 (83)
**Synchronous mets**	Yes No	203 (74) 71 (26)
**Visceral mets**	Yes No	262 (96) 12 (4)
**Lung mets**	Yes No	162 (59) 112 (41)
**Liver mets**	Yes No	62 (23) 212 (77)
**Lymph nodes**	Yes No	205 (75) 68 (25)
**CNS mets**	Yes No	105 (38) 169 (62)
**Adrenal mets**	Yes No	41 (15) 233 (85)
**Comorbidities**	Yes No	133 (49) 141 (51)
**Bone mets**	Osteolytic/Mixed Osteoblastic Missing	163 (66) 84 (34) 27
**Number of bone mets sites**	1–2 3–4 5–10 Missing	125 (47) 83 (31) 57 (22) 9
**Hemoglobin**	≤12 g/dl (women); ≤14 (men) Normal value Missing	85 (50) 84 (50) 105
**ALP**	≥220 U/I Normal value Missing	32 (26) 89 (74) 153
**LDH**	≥300 U/I Normal value Missing	70 (53) 63 (47) 141
**TKI**	Gefitinib Erlotinib Afatinib Osimertinib	184 (67) 43 (16) 46 (17) 1 (0.4)

Clinical and pathological characteristics of patients with bone metastatic EGFR-mutated lung adenocarcinoma. (EGFR, epidermal growth factor receptor; CNS, central nervous system; ALP, alkaline phosphatase; LDH, lactate dehydrogenase; TKI, tyrosine kinase inhibitor).

The median age was 50 years, 171 (62%) were female and 103 (38%) were male. The PS, available in 243 (89%) cases, was 0–1 in 222 (91%) and higher than 1 in 21 (9%) patients, respectively. One hundred eighty-two patients (66%) had a history of smoking habit (present or past). The mutational status, available in 251 patients (92%), was as follows: exons 18 in 14 (6%), exon 19 in 144 (57%), exon 20 in 8 (3%), and exon 21 in 85 (34%) patients. Four patients presented compound EGFR mutations: 1 exon 18 and 21, 3 exon 18 and 20.

Twenty-eight out of 51 available patients (55%) presented T790M mutation.

One hundred eighty-four patients received gefitinib (67%), 46 afatinib (17%), 43 erlotinib (16%). One patient (0.4%) received osimertinib. The median time to TKI exposure was 11,1 months (95% Confidence Interval [CI]: 9,8–12,4).

In 203 patients (74%) BMs were synchronous with diagnosis of advanced NSCLC, while 71 patients (26%) developed bone metastasis during follow-up. In 12 patients (4%) bone was the only metastatic site, 13% had bone plus one metastatic site, and 83% had at least two different metastatic sites in addition to bone. Concomitant visceral metastatic sites were lung in 162 (59%), liver in 62 (23%), lymph nodes in 205 (75%), adrenal glands in 41 (15%), and Central Nervous System (CNS) in 105 (38%) patients respectively.

The burden of bone involvement was available in 265 patients (97%). It was limited (1–2 sites) in 125 (47%), intermediate (3–4 sites) in 83 (31%), and extensive (5–10 sites) in 57 cases (22%). Osteolytic and mixed bone lesions were documented in 163 (66%) cases, the remaining 84 (34%) patients had osteoblastic metastasis. In the minority of patients in which blood parameters were available, hemoglobin levels were often below the normal range and high levels of both ALP and LDH were frequently observed.

### Skeletal-Related Events and Relevant Predictive Factors

**Figure 1:**
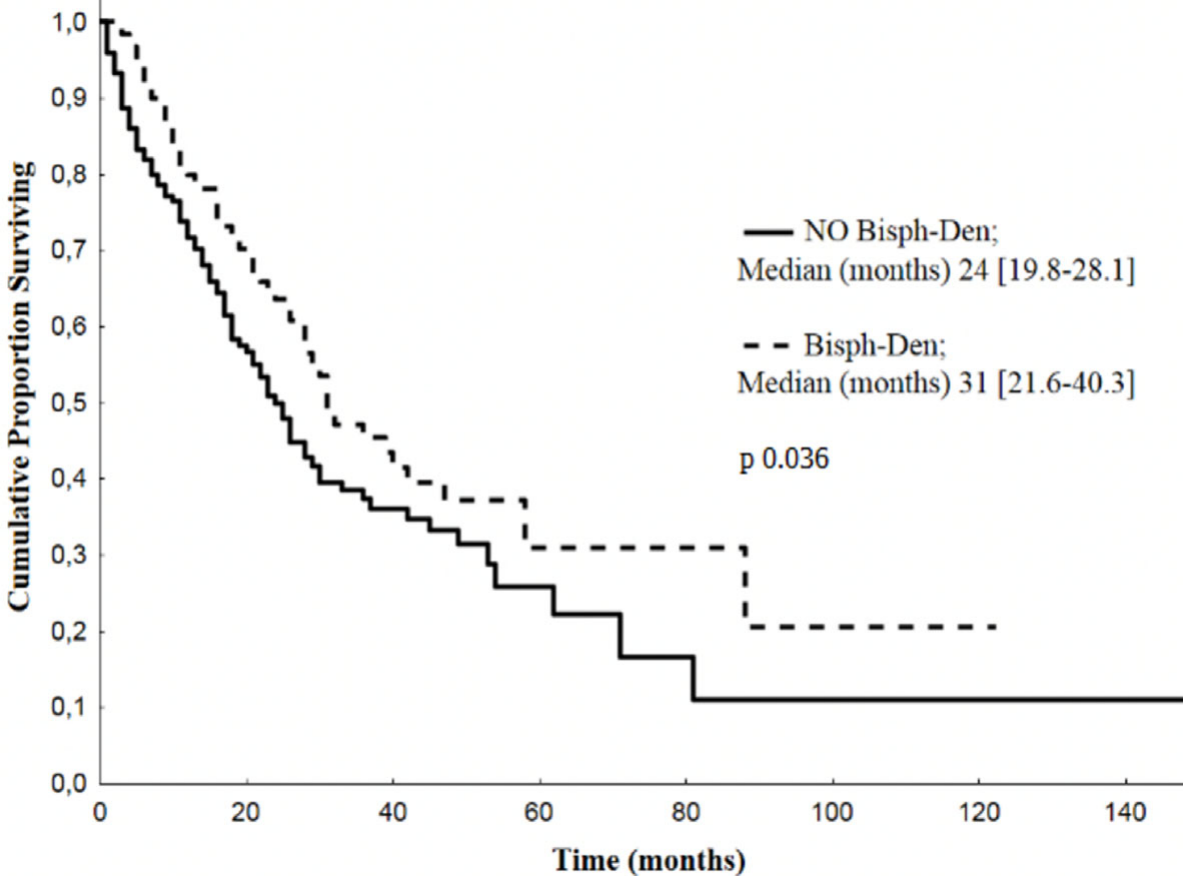
Frequencies of SREs. Percentage and number of global SREs occurred after diagnosis of BM EGFR-mutated lung cancer **(**EGFR, epidermal growth factor receptor; SRE, skeletal related event; BM, bone metastases).

A total of 173 adverse SREs were recorded in the 274 patients examined in the present study (63%). Seventy-seven (28%) developed at least one major SRE: 55/274 (20%) presented bone fractures, 5/274 (2%) hypercalcemia, 30/274 (11%) spinal cord compression. Fourteen patients (5%) developed both pathological fractures and spinal cord compression, three patients (1%) presented hypercalcemia and pathological fractures. Nine patients (3%) underwent bone surgery and 150/274 (55%) radiation therapy on bone (**Figure 1**).

**Figure 2:**
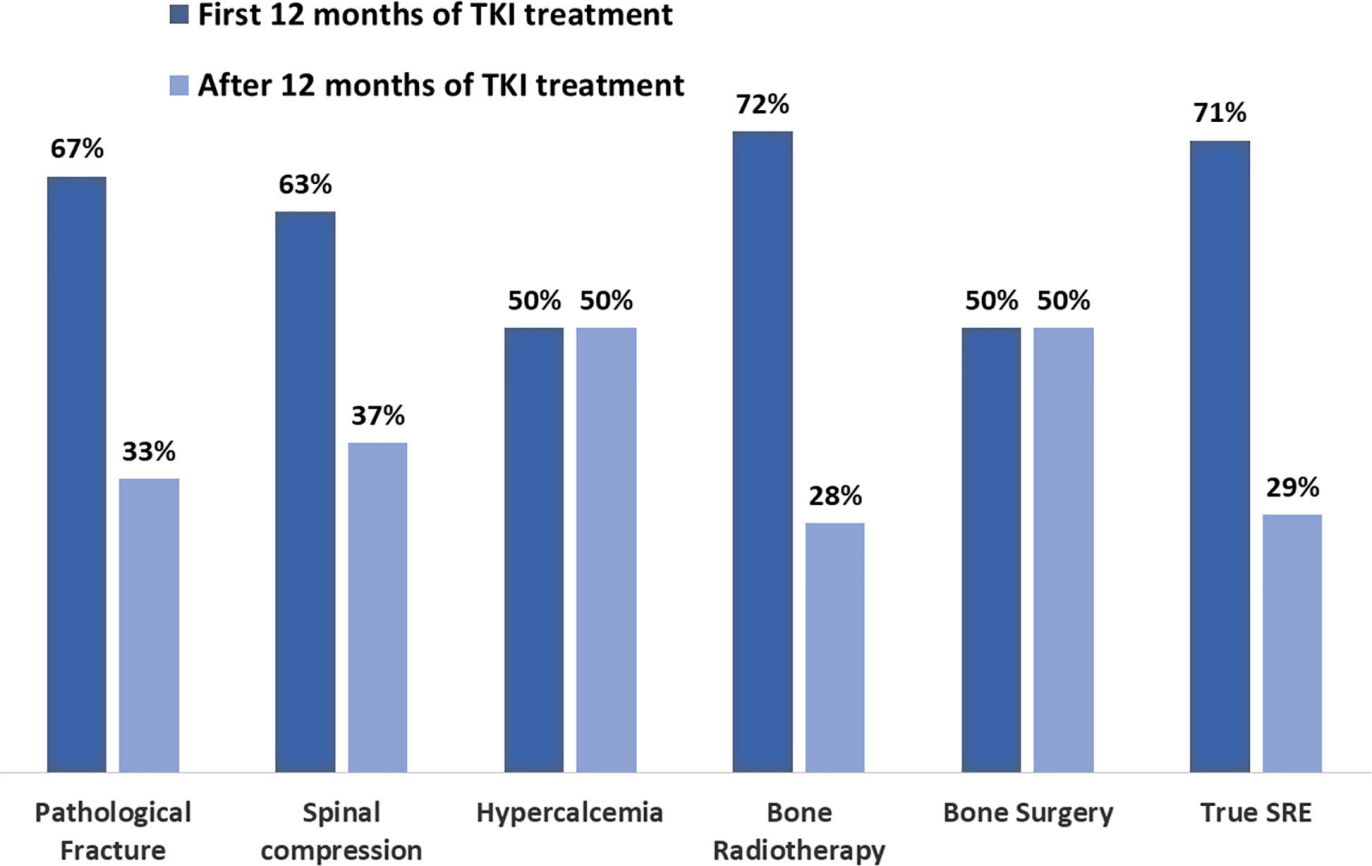
Time to first SRE. Kaplan-Meier estimates of time from diagnosis of bone metastases to onset of first skeletal related event (SRE, skeletal related event).

The date of appearance of the first SRE was available in 135 out of the total of 173 SRE (78%). All the 77 major SREs were correctly placed during the treatment course. As shown on **Figure 2**, SREs occurred early after the diagnosis of BMs. In the patient subset who developed SREs, the median time to the onset of SRE from the diagnosis of BMs was 3.63 months (95% CI 0.79–6.47).

**Figure 3:**
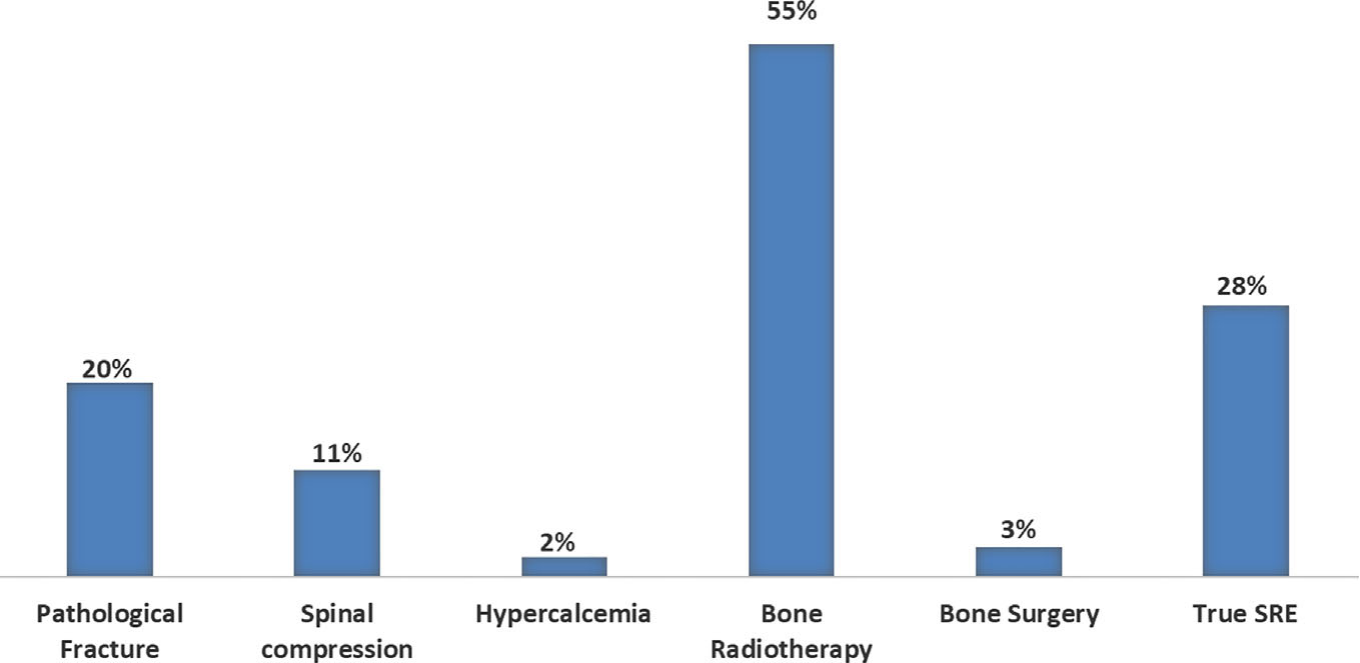
Frequencies of SRE before and after the first 12 months of TKI treatment. Percentage and number of global SREs occurred after diagnosis of BM EGFR-mutated lung cancer before and after the first 12 months of TKI treatment, respectively (EGFR, epidermal growth factor receptor; SRE, skeletal related event; BM, bone metastases; TKI, tyrosine kinase inhibitor).

Forty-one (30%) patients developed the first event before TKI introduction, among the remaining 94 patients, 22/33 (67%) developed bone fractures within the first 12 months of TKI therapy and 11/33 (33%) afterwards; the corresponding distribution of spinal cord compression was 10/16 (63%) and 6/16 (37%), hypercalcemia: 2/4 (50%) and 2/4 (50%), bone surgery: 1/2 (50%) and 1/2 (50%), radiation therapy: 58/81 (72%) and 23/81 (28%), respectively (**Figure 3**).

One hundred and twenty-two patients (45%) received bone resorption inhibitors: 79 (29%) bisphosphonates, 46 (17%) denosumab, 3 patients (1%) received both drugs.

In the univariate analysis depicted in **Table 2**, PS and the presence of liver metastasis were significantly associated with the occurrence of SREs. Both variables maintained their predictive role in multivariate analysis: PS (HR 2.124, 95% CI: 1.046–4.313, p = 0.037), liver metastasis (HR 1.946, 95% CI: 1.169–3.239, p = 0.010).

**Table 2:** Predictive factors of SRE onset.

Time to SRE		Univariate analysis			Multivariate analysis		
		HR	95% CI	P	HR	95% CI	P
Gender	Female Male	0.713	0.451–1.125	0.146			
Age at diagnosis	≥50 <50	0.801	0.399–1.608	0.533			
Smoking status	Yes No/Unknown	1.425	0.893–2.273	0.137			
Performance status (ECOG)	≥2 0–1	2.103	1.037–4.263	**0.039**	2.124	1.046–4.313	**0.037**
EGFR mutation ex 19	Yes No	0.974	0.601–1.580	0.916			
Number of metastatic sites	Only BM 2 ≥3	0.517 0.744	0.127–2.112 0.357–1.551	0.499 0.358 0.430			
Synchronous mets	Yes No	0.963	0.572–1.622	0.888			
Visceral mets	Yes No	1.233	0.718–2.118	0.448			
Lung mets	Yes No	0.931	0.737–1.177	0.550			
Liver mets	Yes No	1.741	1.070–2.833	**0.026**	1.946	1.169–3.239	**0.010**
Lymph nodes	Yes No	1.030	0.612–1.733	0.912			
SNC mets	Yes No	1.308	0.829–2.064	0.248			
Adrenal mets	Yes No	0.676	0.325–1.407	0.295			
Number of bone mets sites	1–2 3–4 5–10	0.671 0.691	0.382–1.178 0.374–1.275	0.337 0.165 0.237			
Hemoglobin	≤12 g/dl (women); ≤14 (men) Normal value	0.976	0.521–1.830	0.939			
ALP	≥220 U/I Normal value	1.725	0.798–3.729	0.166			
LDH	≥300 U/I Normal value	0.921	0.450–1.885	0.821			

Univariate and multivariate analyses of risk factors associated with SRE occurrence according to Cox’s proportional hazards regression model (SRE, skeletal related event). Bold values denote statistical significance at the p < 0.005 level.

### Patient Prognosis and Survival Outcomes

**Figure 4:**
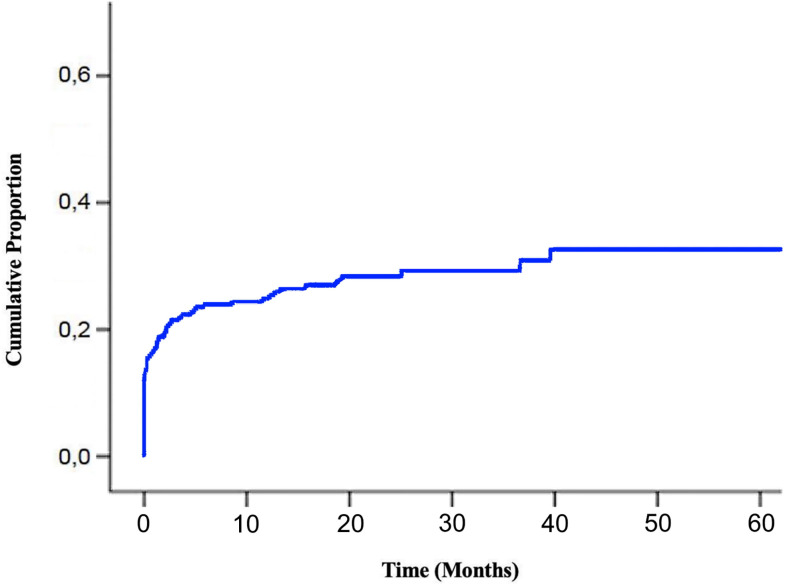
Overall Survival of BM EGFR-mutated patients. Kaplan-Meier estimates of overall survival of EGFR-mutated lung cancer patients after diagnosis of bone metastases (BM, bone metastases; EGFR, epidermal growth factor receptor).

Median follow-up of enrolled patients was 23 months (range 1–117). At the last follow-up examination, 152 patients (55%) were dead. Median overall survival (OS) from the diagnosis of bone metastases was 28 months (95% CI: 24.1–31.8) (**Figure 4**).

Median OS of patients who developed synchronous bone metastases was 29 months (95% CI: 22.8–35.2) as opposed to 24 months (range 15.4–32.6) of patients who presented metachronous bone metastases (p = 0.010).

Among the 33 potential prognostic factors analyzed in univariate analysis, the female gender (HR 0.684, 95% CI: 0.493–0.949, p = 0.023), EGFR Mutation of Exon 19 (HR 0.601, 95% CI: 0.416–0.867, p = 0.007) and the presence of synchronous bone metastasis (HR 0.631, 95% CI: 0.444–0.896, p= 0.010) were significantly associated with a lower risk of death.

On the contrary, smoking habit (HR 1.487, 95% CI: 1.061–2.084, p= 0.021), PS ≥ 2 (HR 1.877, 95% CI: 1.055–3.341, p = 0.032), ALP ≥220 U/I (HR 1.938, 95% CI: 1.147–3.276, p = 0.013), and LDH ≥300 U/I (HR 1.782, 95% CI: 1.126–2.821, p = 0.014) were significantly associated with a higher death risk (**Table 3**).

**Table 3:** Prognostic factors of BM OS.

Overall Survival		Univariate analysis			Multivariate analysis		
		HR	95% CI	P	HR	95% CI	P
Gender	Female Male	0.684	0.493–0.949	**0.023**	0.740	0.514–1.064	0.104
Age lung dg	≥50 <50	1.167	0.645–2.111	0.610			
Smoking status	Yes No/Unknown	1.487	1.061–2.084	**0.021**	1.635	1.134–2.356	**0.008**
Performance status (ECOG)	≥ 2 0–1	1.877	1.055–3.341	**0.032**	2.360	1.296–4.297	**0.005**
EGFR mutation	18/20 19 21	0.601 1.010	0.416–0.867 0.557–1.831	0.014 **0.007** 0.974			
Number of metastatic sites	Only BM 2 ≥3	0.812 0.908	0.356–1.848 0.566–1.458	0.829 0.619 0.690			
Synchronous mets	Yes No	0.631	0.444–0.896	**0.010**	0.481	0.328–0.707	**0.000**
Visceral mets	Yes No	0.865	0.603–1.239	0.429			
Lung mets	Yes No	1.022	0.868–1.203	0.792			
Liver mets	Yes No	0.872	0.724–1.050	0.149			
Lymph nodes	Yes No	0.803	0.567–1.137	0.216			
SNC mets	Yes No	1.335	0.967–1.844	0.079			
Adrenal mets	Yes No	1.102	0.711–1.708	0.664			
Type of bone mets	Osteolytic Mixed Osteoblastic	1.209 1.155	0.827–1.767 0.697–1.913	0.617 0.328 0.577			
Number of bone mets sites	1–2 3–4 5–10	0.892 1.081	0.581–1.371 0.686–1.702	0.594 0.603 0.738			
Hemoglobin	≤12 g/dl (women); ≤14 (men) Normal value	1.229	0.817–1.851	0.323			
ALP	≥220 U/I Normal value	1.938	1.147–3.276	**0.013**			
LDH	≥300 U/I Normal value	1.782	1.126–2.821	**0.014**			
Skeletal related events	Yes No	0.976	0.679–1.403	0.894			
Pathological fracture	Yes No	0.935	0.618–1.413	0.749			
Spinal compression	Yes No	0.986	0.587–1.658	0.958			
Hypercalcemia	Yes No	1.164	0.430–3.150	0.764			
Bone surgery	Yes No	0.584	0.215–1.588	0.292			
Bone radiation therapy	Yes No	1.105	0.794–1.537	0.554			
Denosumab/Bisphosphonate	Yes No	0.704	0.507–0.978	**0.036**	0.722	0.504–1.033	0.075

Univariate and multivariate analyses of clinicopathological prognostic factors of OS in bone metastatic EGFR-mutated lung cancer patients according to Cox’s proportional hazards regression model (BM, bone metastases; EGFR, epidermal growth factor receptor; OS, overall survival). Bold values denote statistical significance at the p < 0.005 level.

At multivariate analysis, smoking habit (HR 1.635, 95% CI: 1.134–2.356, p = 0.008), PS (HR 2.360, 95% CI: 1.296–4.297, p = 0.005), and the presence of synchronous bone metastasis (HR 0.481, 95% CI: 0.328–0.707, p = 0.000) maintained a significant association with the death risk. Due to the low number of patients with available data, LDH and ALP were not included in the multivariate model.

**Figure 5:**
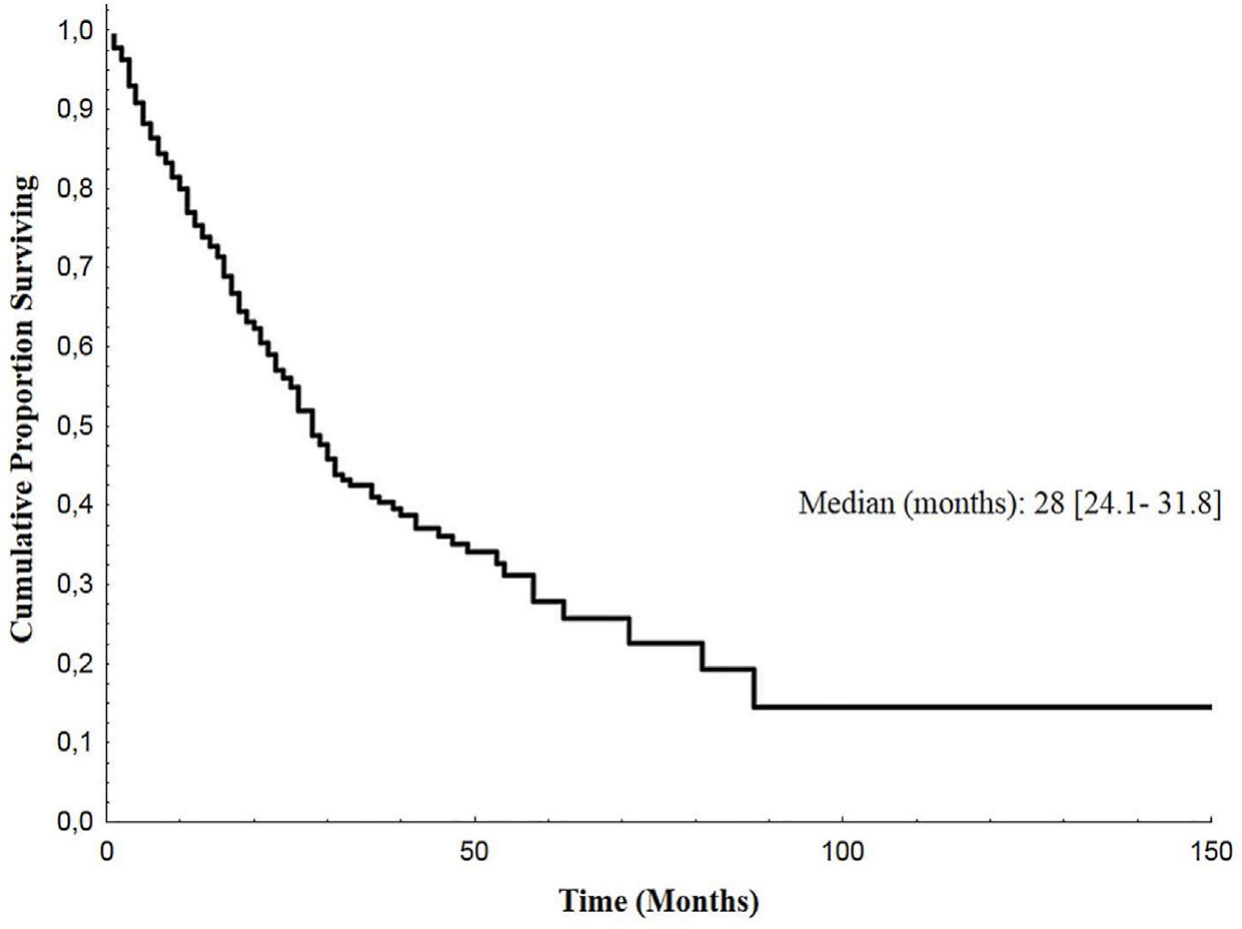
Overall Survival according to administration of bone resorption inhibitors. Kaplan-Meier estimates of overall survival of EGFR-mutated lung cancer patients after diagnosis of bone metastases according to the administration of bisphosphonates/denosumab (EGFR, epidermal growth factor receptor).

The administration of bisphosphonates or denosumab was significantly associated with a lower risk of death at univariate analysis (HR 0.704, 95% CI: 0.507–0.978, p = 0.036) (**Figure 5**) with a tendency to maintain a prognostic significance at multivariate analysis (HR 0.722, 95% CI: 0.504–1.033, p = 0.075).

## Discussion

Bone metastases have a negative impact on quality of life and prognosis of lung cancer patients due to bone pain and the elevated risk of SREs (8). The frequency of SREs in EGFR mutated lung cancer with bone metastases have been scarcely documented in literature (**Table 4**) and the benefit of TKIs on bone pain and SREs prevention have not evaluated in randomized clinical trials (11). The natural history of the disease and frequency of SREs of lung cancer patients with bone metastases have been described in a large number of Italian patients (n = 661) (18). In this series, the median survival after bone metastases diagnosis was 9.5 months and the distribution of major SRE was 16% fractures, 6% spinal cord compression, and 2% hypercalcemia. In this paper, 30% of patients were treated of TKIs, but this subset was not analyzed separately.

**Table 4:** Review of the literature.

Reference	N of BMpts EGFR+	SREsN (%)	Type ofSRE	Antiresorption treatment	Timeto SRE	OS (months)TKI alone	OS (months)TKI+BPH
Cui et al., *Oncol Lett* 2019 (13)	49	23 (47)	–	BPH (32 pts)	–	22	31
Huang et al., *Oncotarg* 2015	62	0 (0)	–	BPH (43 pts)	–	10.4	25.2
Zhang et al., *Sci Rep* 2017 (14)	356	70 (20)	–	BPH (111 pts)	–	19.5	20.5
Nagata et al., *Osaka City Med* *J* 2013 (15)	78	37 (47)	RT 15	–	14.2	–	–
Hendriks et al., *Lung Canc* 2014 (16)	37	19 (51)	–	–	12.9	–	–
Huang et al., *Oncotarg* 2017 (17)	201	75 (37)	RT 39 Surgery 3 Fracture 26 Spinal Comp 7	BPH (57 pts)	–	–	–

Analysis of six papers concerning outcome of patients with EGFR-mutated lung cancer with bone metastases (EGFR, epidermal growth factor receptor; SRE, skeletal related event; BPH, bisphosphonate; OS, overall survival; BM, bone metastases; TKI, tyrosine kinase inhibitor).

In this multicenter retrospective series, involving bone metastatic patients with EGFR mutated NSCLC, the frequency of SREs, according to the standard definition that also includes bone surgery and radiation therapy was 58%. This proportion is similar to that reported in the above mentioned Italian series of bone metastatic lung cancer patients (18), 94.3% of them treated with chemotherapy. Also, the proportion of major SREs (28%), observed in the present study, is similar to that reported in the mentioned series as well as in other published bone metastatic patient series with different primary histologies (19–21). The outcome of patients with EGFR mutated lung cancer with bone metastases have been evaluated in six published papers in which the SRE frequency was 0% in only one paper (22), while ranged between 20 and 51% in the remaining five papers (13–17) (**Table 4**). On the bases of these results, TKIs seem to be not effective in preventing SREs, despite their great efficacy in controlling the disease. This hypothesis is further strengthened by the short time from the diagnosis of BM to the appearance of SRE (3.63 months) observed in our series and the greater distribution of these events in the first 12 months, a period in which the tumor is generally responsive to TKIs.

Preclinical *in vivo* studies demonstrated that EGFR is essential for osteoblast proliferation (23) and down-regulated EGFR signaling was shown to favor the senescence of osteoprogenitors and the decline in bone formation on the endosteal surface of cortical bone (24). The administration of EGFR inhibitors, therefore, could impair the osteoblast mediated bone repair thus favoring the early occurrence of SREs despite the efficacy of these drugs. This hypothesis deserves to be further explored.

Wnt signaling is aberrantly activated in lung cancer (25). Abnormal activation of this pathway is implicated in driving the formation of lung cancer bone metastasis and has a relevant involvement in the cancer induced bone lesions (26). Moreover, Wnt signaling has also a significant negative impact on lung cancer prognosis and therapeutic resistance, specifically as regard as TKI therapy (25). On these bases there is a strong rationale for the testing drug targeting this pathway in association with currently available TKIs, with the aims of prevent bone progression, SREs and overcome/delay treatment resistance (25, 26).

The occurrence of SRE in prostate cancer patients is significantly associated with a poor prognosis (27). This was not the case in this series of TKI treated lung cancer patients. This observation may imply a long deterioration of quality of life in this patient subset in case of occurrence of an SRE.

The overall survival of 28 months, observed in this patient series does not differ from that observed in clinical trials and published case series (3–5), so the presence of bone metastases in TKI treated patients seem to be not related to poorer prognosis. The prognostic factors observed in univariate and multivariate analyses (i.e. ECOG Performance Status, smoking habit, and synchronous bone metastasis) in this study are in line to those observed in published series (3–5).

Several randomized prospective clinical trials have demonstrated that bone resorption inhibitors are efficacious in preventing SREs of bone metastatic lung cancer patients (10, 12). These trials were conducted before the introduction of TKIs in clinics, so we do not have a formal demonstration of their efficacy in this clinical setting. In the present series, these drugs failed to correlate with a lower SRE proportion, due to their delayed administration (i.e. after the onset of the first SRE) in many cases. The great proportion of SREs observed in the present study and their early onset provide a strong rationale for the introduction of bisphosphonates and denosumab in this setting. Interestingly, the administration of these drugs was associated with better prognosis, that just failed to attain the statistical significance in multivariate analysis. A positive effect of bisphosphonate administration on survival of EGFR mutated lung cancer patients submitted to TKIs was previously observed in 3 published series (13, 14, 22) (**Table 4**).

Despite the recommendation of using anti-bone reabsorption agents, only 45% of our population received these drugs and most of them start treatment after SREs onset. The reason could be related to the fact of TKI are expected to very efficacious in this setting. At last but not at least physicians are afraid of the possible occurrence of jawbone osteonecrosis, one of the most severe adverse reactions.

Bisphosphonates could enhance the antineoplastic effects of EGFR-TKIs in NSCLC with EGFR mutation both *in vitro* (28) and *in vivo* (14). Pertinently, the results of a randomized clinical trial of denosumab versus zoledronic acid in patients with various primary malignancies have shown a survival advantage favoring denosumab in the subset of patients with NSCLC (29). On the bases of this background, the synergism between bone resorption and EGFR inhibitors in lung cancer patients deserves to be explored.

The retrospective nature and the absence of a post progression analysis are the main limitations of this study.

## Conclusions

In conclusion, bone metastatic NSCLC patients with EGFR mutated disease, treated with modern EGFR inhibitors, have a relatively long survival expectancy and are at high risk to develop skeletal related events. Since SREs occur early after the TKI start, there is a rationale to administer bone resorption inhibitors. Whether bisphosphonate or denosumab have the potential to improve survival when associated to TKIs is a matter of future research.

## Data Availability Statement

The raw data supporting the conclusions of this article will be made available by the authors, without undue reservation.

## Ethics Statement

The study was firstly approved by the Institutional Review Board of the Coordinating Center in Brescia (SURMOS Study no. NP1848) and subsequently by the ethic committees of each participating institution.

## Author Contributions

All authors contributed to the study design and data collection for all the Italian institutions involved in the study. Material preparation and analysis were performed by ML and CG. The first draft of the manuscript was written by ML, CG, and AlfB. All authors contributed to the article and approved the submitted version.

## Conflict of Interest

The authors declare that the research was conducted in the absence of any commercial or financial relationships that could be construed as a potential conflict of interest.
